# Through the Looking Glass: Parental Group Experiences Observing Sensory Motor Therapy

**DOI:** 10.1155/2018/2468037

**Published:** 2018-10-17

**Authors:** Joan Vertes, Chrystelle Robinson, Veronica Gershenzon, Emily S. Ho, Ashlee Vennettilli

**Affiliations:** ^1^Department of Occupational Science and Occupational Therapy, University of Toronto, Canada; ^2^Department of Rehabilitation Services, The Hospital for Sick Children, Canada; ^3^Department of Rehabilitation Services, Division of Plastic and Reconstructive Surgery, The Hospital for Sick Children; Rehabilitation Sciences Institute, University of Toronto, Canada

## Abstract

**Objective:**

The benefits of group therapy in pediatric rehabilitation have been identified. However, a unique small group occupational therapy model with a large emphasis on parental group education and observation of their children has not been extensively studied. In this model, parents observe their child's sensory motor group therapy through a one-way mirror and work with the occupational therapist together after each session, to receive education and develop strategies. In other models, parents sit in the waiting room or observe without working with an occupational therapist as a group afterwards.

**Method:**

A descriptive qualitative study was conducted to explore the parental experiences of observing and receiving information as a group regarding their child's participation in sensory motor group therapy. Individual in-depth interviews were conducted with ten parents who observed their children together through a one-way mirror during their children's therapy. Conventional thematic content analysis was used to analyze the interview transcripts to determine themes. Ten parents were interviewed.

**Results:**

There were three major themes that surfaced: *parent support*, *the value of observation*, and *knowledge*. Parents enjoyed and perceived benefits for themselves and their children from the opportunity to observe sessions and receive information as a group during therapy.

**Conclusion:**

The experiences of parents in this group model suggest that knowledge translation and provision of support to parents and their children regarding their sensory motor needs are beneficial. Administrators may appreciate additional gains of reducing costs and improving access to service.

## 1. Introduction

### 1.1. Pediatric Rehabilitation Group Therapy

Literature reports that approximately half of occupational and physical therapists utilize some group models of practice [1, 2]. Little has been written specifically about occupational therapist coupling group work with children, as well as separately with their parents in groups.

Various types of occupational and physical therapy groups have been described, such as sensory motor [3], feeding, and social skill groups [4]. The benefits and challenges of a group model of practice in pediatric rehabilitation have been documented to a limited degree. Camden et al.'s study [1] of perceived efficacy of groups according to interviews and focus groups with administrators, therapists, and parents revealed that from a health system perspective, both groups increase service accessibility at the same time as promoting positive therapy outcomes. LaForme Fiss and Effgen [2] conducted a survey with pediatric therapists and found that among the 285 who were polled, 41.4% were reported using groups in their practice. However, half felt that group therapy was “not effective” or only “somewhat” effective in meeting goals. Thirty-nine percent of the respondents felt it was not as effective as individual sessions. The authors recommended further research to examine the effectiveness of groups in clinical settings.

### 1.2. Family-Centred Care

Collaborating with parents is a tenet of family-centred care. This model is a common pediatric occupational therapy (OT) approach that emphasizes parent-therapist collaboration in intervention [5, 6]. Providing family-centred care requires that therapists understand and consider parental hopes for therapy for their children and work together with both the child and the family to achieve goals [5]. Previous qualitative research exploring parents' experiences with sensory motor OT suggests that parental hopes for results fall into two categories: child-focused outcomes and parent-focused outcomes [5]. Parents' overwhelming wish is that the OT experience could provide them with personal validation (support for their parenting strategies, understanding of their challenges, and reassurance that their child is not weird and that they have real problems) and strategies to support their child (resources, information, and techniques to support development).

A family-centred approach providing sensory motor therapy recognizes that parents need and want support. Previous research has been conducted on the parental experiences of waiting together during their child's OT treatment sessions which showed that a “waiting room phenomenon” occurred [7]. Simply put, parents reported that sitting together in the waiting room enabled them to exchange information, resources, and strategies, as well as provide social support to one another. This experience also allowed parents to reframe their views and expectations of their children's abilities and challenges. In a companion study by Cohn [8], parents believed that this reframing allowed them to become more accepting of their children, which they believed would contribute to an improved sense of self-worth in their children.

Research on the use of groups in pediatric rehabilitation is informative for clinicians. However, no studies reviewed to date described a group model whereby parents received education and support together as a group from occupational therapists, following each session. Exchange of knowledge and experience between therapists and families can indeed foster collaboration that optimizes results in therapy [9]. Vertes et al. [3] evaluated the benefits of both educating parents together and providing them with the opportunity for group interaction.

Program evaluation indicated that parents found watching the group with others to be beneficial [3].

It is important to delve into the experiences of participating parents to further understand the components and characteristics of this group model that contributes to its perceived benefits. Therefore, the purpose of this research is to explore parental experiences of observing and receiving information as a group regarding their child's participation in sensory motor group therapy.

## 2. Methods

### 2.1. Sensory Motor Group Therapy with Parental Involvement

In recognizing the benefits of group therapy for both children and parents, a small group OT model was developed and implemented at the authors' institution for children between 4 and 12 years of age with difficulty processing and integrating sensory information. They may be referred with sensory motor difficulties, learning disability, developmental coordination disorder, attention deficit disorder, and/or writing problems. Their performance may be affected in many ways in school, at home, or in the playground: e.g., poor handwriting, difficulty in gym class, sensory hypersensitivity and irritability, poor attention and concentration in class, low self-esteem, and/or social skill challenges. Goals for the children include improving motor skills, teaching them about sensory issues and strategies to deal with them, and developing communication and social skills. Groups typically contain four to five children of similar ages with two occupational therapists and run on a weekly basis. They are one-hour in length and provided in blocks of seven to eight sessions. Sessions include “a sequence of warm-up and organizing activities, such as (a) gym ball, scooter boards on ramps, and/or theraband; (b) games on suspended equipment (swings); and (c) a combination of fine and visual motor activities while at the table or blackboard” ([10], p. 5).

Parents watch together behind a one-way mirror during their children's sensory motor OT. Following sessions, parents meet with the occupational therapists as a group to receive education and discuss each of their children, reviewing activities, abilities, and behaviors observed, for 30 minutes. Parents are taught how individual sensory motor issues may affect occupational performance, and recommendations are explored for school and home. They have the opportunity to ask questions and share resources with one another and occupational therapists. Private discussion is provided when the parent and/or therapist wishes, where additional 30 minutes are offered. Most parents are comfortable sharing questions and concerns together, but some ask to discuss specific issues privately. An OT individual report is reviewed with each parent in a one-hour final review session following the completion of the block of therapy.

### 2.2. Procedures

This research was first approved through the two Research Ethics Board in accordance with the procedures at the respective authors' institutions. A descriptive qualitative research study was conducted using individual in-depth interviews to gain an understanding of the social and personal aspects of each participant's experiences [11]. Conventional content analysis was employed to derive meaning from parent interviews. This process involved the subjective interpretation of the content of text data through systematic coding and identifying themes or patterns [12]. Research using this approach focuses on communication through language, paying attention to the meaning of the text with the goal of providing understanding of the phenomenon under study [12].

### 2.3. Participants

A purposeful sample of participants was recruited from a pool of clients who attended sensory motor therapy during the two most recent therapy sessions. Parents were contacted via telephone after the completion of the therapy to be invited to participate in the study. As per institutional ethics protocol, study participants must be recruited by an individual within their circle of care. Thus, parents were contacted by the first author, who was their children's treating occupational therapist. Parents were invited to participate in the study if they had observed their child participate in therapy along with other parents, if they had attended at least five sessions, and if they could communicate with at least a conversational level of English. There were nine mothers and one father who participated in the study. This was representative of the gender of the caregivers who typically attended sensory motor therapy with their children. Recruitment of participants stopped when theoretical saturation of codes was achieved, defined as consensus among the researchers that there was sufficient repetition of codes, indicating a depth of information to support the major themes, as suggested in the literature on qualitative methods [13].

### 2.4. Interviews

In-depth interviews were conducted by two of the researchers, both student occupational therapists, who were not involved in the sensory motor therapy or the participant recruitment process for this study. The interview guide (Appendix A) was developed collaboratively by the researcher team members which contained open-ended questions that were designed to explore parental experiences. Probes were used to encourage participants to provide more information [14]. Interviews ranged from 30 minutes to an hour depending on the length of participants' responses. On request, the interviewers accommodated three of the parents by conducting the interviews at their homes. All others were conducted at the authors' institution. The interviews were audio recorded and transcribed verbatim by the researchers and a research volunteer who was a medical student who was not part of the investigative team. Interviewers also recorded observational notes in a chart format for each interview describing the environment and the participant's nonverbal cues such as body language, the tone of voice, and apparent comfort level to enhance the meaning of the audio data [14]. All transcripts were checked the second time for accuracy by the two interviewers. Two pilot interviews were conducted. Piloting allowed the interviewers to practice their interview skills and determine whether changes are needed to be made to the interview protocol [15]. Since no major changes were necessary to the interview protocol and informed consent procedures were carried out, the pilot interview data was included in the analysis.

### 2.5. Data Analysis

Once the transcription of the interviews was complete, three investigators independently conducted an open coding process of the text for reasons of trustworthiness and accuracy and to acknowledge researcher bias [15]. Each investigator used the same technique of analysis, coding the text twice from two different points of view: (a) coding for events, actions, and interactions and (b) coding for meanings, feelings, and relationships [15]. The coding process entailed highlighting sections of the texts based on the idea or meaning that arose from the data. Once the individual coding process was complete, the preliminary codes were reviewed by the research team for consistency of coding. Expert checking was conducted by the fourth investigator who reviewed the codes to substantiate the interpretation of the data [16]. This investigator has over 30 years of experience with working with parents of children with sensory motor concerns. The data were reviewed yet again to connect and identify relationships between codes. Then, common themes were derived with consensus by all researchers through several face-to-face discussions. In addition to expert review and peer review, methods taken to address rigour and trustworthiness in this study as described by Taylor and Gibbs [15] included personal reflexivity (i.e., as the key analytical tool, the researchers contemplated where their understanding and interpretations arose from) and an audit trail of the coding process [17].

## 3. Results

The results of this study are thematically represented. There were three overarching themes that emerged: *parent support*, *the value of observation*, and *knowledge*.

### 3.1. Parent Support

Parents reported that the experience in the observation room was akin to a support group. They described developing relationships in a safe environment through the commonalities of raising a child with sensory motor issues. They no longer felt alone and discussed their similar situations and experiences.

“If you have individuals who are similarly invested in sharing, then you feel supported and safe in sharing your experiences and challenges.” “So there's something that's positive about not only gleaning pragmatic resource type of information but also more of an emotional ‘I'm not alone in this'…,” and “…comfort in knowing that I wasn't the only one and that I had people I could talk to and who really understood …”

Parents unanimously spoke of the ability to share and receive resources. This included strategies, tools, and toys to use at home, extracurricular and leisure activities to help promote development, and services and programming in the community. This support was obtained from group members and therapists, both during observation time and with therapist afterwards.

“We now have a toolkit of activities to help with his sensory motor difficulties.” Parents merged their individual learning and personal experiences and related them to one another's situations: “…we're also able to share resources… I was able to get a sense of how other families found ways to cope with different kinds of behaviors or different kinds of sensory stressors.”

They could now cope better with their children's difficulties: “It helps that other people are giving their inputs about their children's experiences and what works for them, so that you're sitting there and you're going oh maybe I should try that one and see if it works for x. So it's good to have other people with their experiences as well.”

### 3.2. The Value of Observation

The data suggested that having the opportunity to observe their child's therapy was an important part of the parents' experience. It was found to be helpful to watch and compare their own child with others in the group.

“...the opportunity to actually watch your child through this glass is far more beneficial… you're part of the therapy.” “…It was great because it allowed me to see not only how my child was progressing...it's completely different to see firsthand what's going on… you can see how they're interacting with other kids…I guess also to know in the grand scheme where he was at compared to his peers.”

There was strong consensus that observing enabled parents to understand their child's difficulties and how to help them. They could better appreciate their child's sensory motor issues, treatment progress, and benefits of the group therapy approach.

Watching allowed the parents to appreciate the intricacies of sensory motor therapy. They directly observed how the occupational therapist modified and tailored each session according to the individual needs of the children. They felt they were better equipped to continue the therapy at home and help their child. “I can copy techniques used in class (OT) at home.”

Parents commented on the skills of the occupational therapists, the group therapy design, and how much the children enjoyed partaking in therapy. “He learned a great deal in the best possible way, by having lots of fun!” “And we'd always discuss it on the way home, we'd discuss everything he did, and I was able to do that because I was watching.”

The one-way mirror also provided the opportunity to address the parents' great concern with their child's social skills. Parents expressed joy in observing their children engage in constructive interactions with other children; e.g., children helping one another during challenging tasks. Several parents commented on the advantage of watching their children in a way that did not cause them embarrassment. Parents noted how their children behaved with peers. “The most important thing is that they did not see us. They knew we were there but they didn't know what we're doing. In my case, x is afraid of making a mistake in front of us because he doesn't want to let us down. The simple fact that he doesn't see us looking at him gave him the freedom.”

Parents expressed that they did not feel like they were “waiting” for their child to finish the session, because of the active observation component and the ability to talk to the other parents. “It didn't feel like a waiting room… But here it felt like we were watching our kid perform...They'd always pull things, you know, and I wasn't bored, I enjoyed it.”

### 3.3. Knowledge

Feedback with the occupational therapists was reported to be beneficial. Knowledge was enhanced. “We're understanding how she processes things and so can cope better with frustrations”; “we understand his need for stimulation.”

“The therapist's feedback was both collaborative and individual—very helpful!”; “his teachers use many of the therapist's recommendations…they were skeptical at first…but had to admit they saw an improvement and encouraged us to continue!”

Parents valued discussing their children's' participation as well as how activities were adapted for individuals. They developed a better understanding of their children's sensory motor issues and how to apply various therapeutic approaches and tools. “Talking to staff, getting the advice meant a lot.” “It increased knowledge and ideas to cope.” “I have a better understanding of how we can help him.” “I now have specific tools for my child's unique issues.” “I thoroughly understand SPD (sensory processing disorder) now.” “Better able to understand his problems with motor planning.” “It was great to learn and feel understood by Joan (OT) and parents in the group.”

Parents had the opportunity to ask specific questions about their child or learn from others and the occupational therapist. One parent in this study described how the group helped her to brainstorm methods to obtain greater support from her child's school principal regarding a particular issue. Parents reported that their anxiety regarding difficulties was often reduced and their optimism increased. “I am learning that some of his quirks are not deliberate.” “I have a better understanding of what challenges he has.” “I have decreased fear of his problems related to a more serious pathology as CP (cerebral palsy), MD (muscular dystrophy).” “It opened my eyes to x's needs and a more optimistic view of the future.”

Changes in their children's' participation were recognized: “He has increased confidence in participating in physical activities, trying new ones; won't Joan (OT) be proud of me. Tried wakeboarding…” “We recently bought a kayak and he really took to it….he may not be in the Olympics but felt positive and really took to it.”

Positive feedback regarding knowledge and parent support obtained was reflected by many parents who asked if they could return for another therapy block or continue to stay in touch. “I suggest a chat group on internet where we could post stuff for one another and ask the OT questions.” “Very positive and helpful sessions—first significant help we have had for our son. Really want to come back again!”

## 4. Discussion

This qualitative study was conducted to address a gap in the literature regarding the impact of a family-centred care group model. Although several studies describe the value of using groups, none have examined parents' perspectives of a model where they observe their children's sensory motor therapy group through a one-way mirror with the opportunity to discuss observations, concerns, strategies, and recommendations together with the occupational therapist afterwards. In depth interviews were conducted. Three themes related to parent perceptions emerged from this investigation, including parent support, the benefit of observation, and knowledge.

### 4.1. Parent Support

Parents within the naturally formed support group engaged in processes of validation and support with one another, as well as with the occupational therapist. They shared information and resources and were relieved to find that they were not alone and that others had similar concerns or issues. They described being better able to cope when the feeling is thus supported. This theme aligns with a similar one found in the study by Cohn [7] where parents shared information and resources and felt supported by other parents in coping with their children's challenges. These findings support the benefit of establishing an environment where parents can be together during their children's therapy visits. This notion is further supported by LaForme Fiss' [4] description of the value to parents of sharing with peers. Interestingly, while Cohn [7] described a “waiting room phenomenon,” parents in our study did not feel the experience was akin to waiting because of the ability to observe the therapy. The authors believe this observation room setting provided parents with a more participatory role in their child's therapy.

### 4.2. The Benefit of Observation

The one-way mirror was found to be a fundamental aspect of this group experience. Parents expressed many benefits of being able to observe therapy. They observed changes and progress made by their children in therapy. They commented on how activities were modified and tailored to their children's' individual needs. Parents were able to observe how their children interacted with others socially. The children could be themselves since they did not feel they were being watched. Parents appreciated how having fun when learning was an important part of therapy. They described being excited to copy therapists' techniques and ideas after observing, to better help their children.

### 4.3. Knowledge

Knowledge was provided and highly appreciated. Parents were enthusiastic in discussing their observations together with the occupational therapists following each session. They reported a greater understanding of their child's sensory motor difficulties, how these may have been contributing to frustration, and ways to continue therapy at home, something they were not always able to gain from regular therapy sessions that do not allow parent participation (waiting rooms). Parents described having diminished anxiety and greater optimism as a result of this approach. Understanding may have been enhanced by providing a setting where parents could discuss what they observed with one another and therapists.

Parents learned by receiving information from the occupational therapists about what was observed, while gaining further insight from feedback given to and questions raised by other parents. As such, while parents learned from watching their children and receiving feedback from the therapists, the presence of the other parents served as an added avenue for learning.

### 4.4. Mutual Aid Theory

The current literature points towards the benefit of bringing parents together during their child's treatment session. This phenomenon is supported by mutual aid theory, which can be used as a guiding theoretical framework for therapists to explore the benefits of parent groups. Shulman [18] described groups as a form of “mutual aid” where people work together with a therapist on common issues, to help each other. “…each member can contribute to the common pool of knowledge. The leader will also contribute data which, when combined with that of the others, provide a rich resource for the members” [19]. There are nine processes of mutual aid, many of which were reflected in this study: sharing data, the dialectical process, exploring taboo subjects, “all in the same boat” phenomenon, emotional support, mutual demand and expectations, helping with specific problems, rehearsal (i.e., implementation of lessons learned from mutual discussion to everyday life), and strength in number phenomenon. Mutual aid can be used as a guiding theoretical framework for therapists to explore the benefits of parent groups [19].

### 4.5. Mutual Aid Processes and Study Themes

The themes found in this study are in line with the existing literature that suggests groups tend to create a process of mutual aid. Five processes in particular align with study themes. (1) Sharing data refers to how members of a group share resources and ideas they have used to help them cope with similar issues. Similarly, in the parent support theme, the parent group benefitted from actively engaging in the sharing of information (data) with one another and later with the OT. The resources shared pertained not only to individual sensory motor strategies but also to community resources and contacts. (2) All in the same boat refers to how members feel relieved to find that others experience similar concerns, feelings, and problems. Shulman [18] describes a deepening of interactions among individuals who share similar struggles. As described above in the parent support theme, this process was fostered through the study parents' realization that their problems were not unique, but shared by others. There was comfort in knowing that they were not alone. They described feelings of relief and reduced anxiety in the parent support theme. These were further reinforced by being able to watch, as described in the value of observation theme, where they were happy to be able to compare their children to others who were struggling in similar manners. (3) Emotional support refers to how providing empathy can help as much as receiving it. Peer support provided in small groups may be more helpful than staff support. In this study, within the parent support theme, parents described feeling reassured and safe to share with others who were similarly invested and reported feeling better to able to cope. They received empathy from each other and therapists and shared positive strategies. (4) Helping with specific problems refers to how participants help themselves while helping others with a specific problem. An example of this within the knowledge theme of this study was described by one parent who received help from the group in dealing with the child's school principal. (5) Mutual expectations refer to how participants are aware that they are expected to report back to each other following sharing of advice and recommendations [18]. Parents in this study within the knowledge theme described benefitting from hearing one another's and the therapists' ideas and recommendations, looking forward to trying new approaches. They were able to follow up in subsequent sessions.

### 4.6. Limitations

There are a few limitations to this study. The recruiting investigator of the participant sample was also the treating occupational therapist. This method of recruitment had the potential to introduce some bias as it is possible that parents would have wanted to be seen as positive about the program. However, the parents were interviewed by student occupational therapists who had no previous involvement in the therapy. The data were also deidentified before the triangulation stage of the data analysis, and reflexivity was used as methods of ensuring trustworthiness of the qualitative analysis. Deidentification was used to overcome the recruitment limitation as parents were told they would be anonymous and therefore could speak freely.

## 5. Conclusion

In summary, this study provides a novel assessment of group therapy through in-depth parent interviews. Findings suggest that a novel group approach that allows parental observation of their child's sensory motor group therapy through a one-way mirror coupled with detailed parental group education sessions with the occupational therapist is highly regarded by parents. It is a model program where the OT may provide support, therapy, and knowledge to parents and children at the same time. This not only adds to the current literature on the use of group therapy in pediatric rehabilitation but also suggests a need for further research as to the advantages of groups and describes a model for similar programs. These positive findings can provide valuable information to healthcare providers when striving for family-centred and evidence-based practice. Specifically, the findings provide support for the involvement of parents in their children's therapy by providing opportunities to observe and receive information from therapists while being in a group with other parents.

## Data Availability

The transcribed interview data used to support the findings of this study are restricted by the SickKids Research Ethics Board in order to protect patient and family privacy. Data are available from Joan Vertes (joan.vertes@sickkids.ca) for researchers who meet the criteria for access to confidential data.

## Appendix

### A. Interview Guide

1. Describe your experience of being in a group with the other parents. What was it like?
  - What was it like waiting as a group with the other parents?
  - What was it like watching your child as a group with the other parents?
  - What was it like receiving the OT education as a group with the other parents?
2. Can you share with me things you liked or disliked about waiting with other parents while your child received sensory motor group therapy?
3. Can you share with me things you liked or disliked about watching with other parents while your child received sensory motor group therapy?
4. Can you share with me things you liked or disliked about receiving education by the OT as group with other parents?
5. Have you experienced other ways for your child to receive sensory motor therapy or OT?

*General Probe used for all questions: Can you tell me more about this? Can you give me an example? Can you elaborate on this?*
